# The Homocoupling Reaction of Aromatic Terminal Alkynes by a Highly Active Palladium(II)/AgNO_3_ Cocatalyst in Aqueous Media Under Aerobic Conditions

**DOI:** 10.3390/molecules21050606

**Published:** 2016-05-10

**Authors:** Mengping Guo, Bo Chen, Meiyun Lv, Xiuling Zhou, Yongju Wen, Xiuli Shen

**Affiliations:** 1 Institue of Coordination Catalysis, College of Chemistry and Bio-Engineering, Yichun University, Yichun 336000, China; chenbo25719@163.com (B.C.); lvmeiyun2005@163.com (M.L.); 13879593114@163.com (X.Z.); wenyongjujuju@163.com (Y.W.); shenxiuliwyj@163.com (X.S.); 2 Engineering Center of Jiangxi University for Lithium Energy, Yichun University, Yichun 336000, China

**Keywords:** aromatic terminal alkynes, palladium(II)/AgNO_3_ cocatalyzing, homocoupling reaction

## Abstract

A new and efficient Pd(II)/AgNO_3_-cocatalyzed homocoupling of aromatic terminal alkynes is described. Various symmetrical 1,4-disubstituted-1,3-diynes are obtained in good to excellent yields. This protocol employs a loading with relatively low palladium(II) in aqueous media under aerobic conditions.

## 1. Introduction

**Figure 1:**
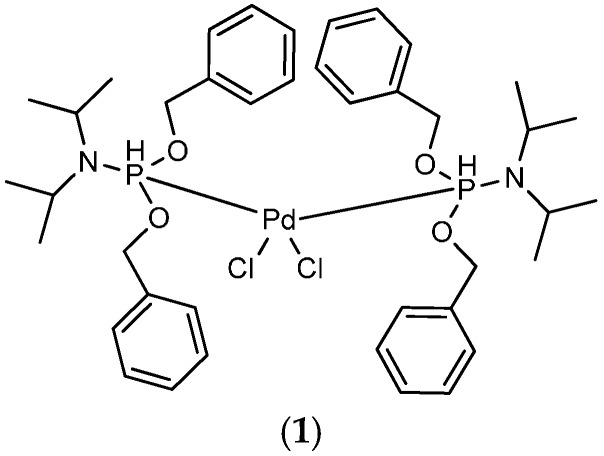
Palladium(II) complex (**1**).

Compounds which contain a 1,4-disubstituted-1,3-diyne linkage have been found in applications in areas ranging from pharmaceuticals with anti-inflammatory, antibacterial, antitumor, and antifungal activities to a large variety of polymers, biologically active molecules, and supramolecular materials with appreciable photoelectrical properties [1,2,3,4,5,6]. The homocoupling reaction of terminal alkyne is the classical protocol, and the choice of the catalytic system is a pivotal factor for the synthesis of symmetrical 1,3-diynes [7,8,9,10]. Palladium complexes in combination with Cu salts are traditionally employed as cocatalysts for the reaction [11,12,13,14,15,16,17,18]. Recently, apart from copper salts, catalysts, based on other transition metals such as Ni [19], Co [20], Au [21], and Ti [22] have also been employed in terminal alkyne homocoupling reactions. Moreover, an iron in combination with a Cu salt cocatalyst has also been reported [23]. Furthermore, the metal-free cocatalyst palladium-catalyzed homocoupling reactions have been described [24,25]. However, to the best of our knowledge, Ag_2_O has been reported as a cocatalyst for the homocoupling reaction of terminal alkynes [26], but Ag(I) as a cocatalyst has not been reported. Our group has recently synthesized and characterized a new palladium(II) complex (**1**) (Figure 1) via single-crystal X-ray crystallography. Owing to the inertness of the palladium(II) complex (**1**) towards oxygen and moisture, it has been used as catalyst in an aerobic Suzuki coupling reaction [27] and an aerobic cyanation of aryl halides [28]. Herein, we wish to report the use of a new Pd(II)/AgNO_3_ cocatalytic system for the homocoupling reaction of aromatic terminal alkyne under aerobic conditions.

## 2. Results and Discussion

### 2.1. Optimization of the Homocoupling Reaction Conditions

In our initial experiments, we observed that the homocoupling of phenylacetylene (1 mmol, **1a**) in the presence of Pd(II) complex **1** (0.5 mol %) and NaOH (1 mmol) in THF/H_2_O (in 4:1 proportion, 2.5 mL) at 60 °C proceeded to give the desired homocoupling product (**2a**) in a small isolated yield (<10%) (Table 1, Entry 1). After an addition of AgNO_3_ (0.05 mmol), the yield was up to 51% under the same conditions (Table 1, Entry 2). This observation prompted us to further investigate the effect of a base on this protocol, finding that the best result was obtained in the presence of Cs_2_CO_3_ as a base (Table 1, Entry 11). The effect of the solvent in the reaction was also studied using a different co-solvent, and a 93% yield of the desired product (**2a**) was isolated in *n*-butyl alcohol/H_2_O (in 4:1 proportion, 2.5 mL) (Table 1, Entry 22). Consequently, *n*-butyl alcohol/H_2_O was chosen as the best co-solvent.

We continued to examine the influence of the ratio of co-solvent, temperature, time, and amount of catalyst and AgNO_3_ on the yields. As can be seen in Table 2, the reaction proceeded perfectly to obtain 97% yield in *n*-butyl alcohol/H_2_O (in 1:2 proportion, 3 mL) in the presence of **1** (0.5 mol %) and AgNO_3_ (0.05 mmol) at 60 °C under aerobic conditions (Table 2, Entry 5), but only a trace yield of 1,4-diphenylbuta-1,3-diyne (**2a**) was obtained in the absence of Pd(II) complex catalyst **1** or AgNO_3_ (Table 2, Entries 7,11). However, when 0.5 mol % Pd(II) complex catalyst **1** and 0.075 mol % AgNO_3_ were used as a cocatalyst, the yield obviously enhanced, with the homocoupling of phenylacetylene (**1a**) being quantitative (Table 2, Entry 14). These results show that both Pd(II) complex catalyst **1** and AgNO_3_ play important roles in the oxidative homocoupling reaction of terminal alkynes. Then the temperature effects were examined in this homocoupling reaction, finding that the homocoupling product **2a** was obtained in higher yield (99%) at 60 °C, but in lower yield (92%) at 80 °C (Table 2, Entries 14,21), which is consistent with the results of Shi and colleagues [14]. With a lower temperature (40 °C), **2a** was obtained in moderate yield (56%) (Table 2, Entry 20). Under these reaction conditions, prolonging the homocoupling reaction time from 4–24 h, the yield of **2a** increased from 13% to 99% (Table 2, Entries 14,16–18). In a word, the best result was obtained to carry out the reaction in *n*-butyl alcohol/H_2_O (in 1:2 proportion) using Cs_2_CO_3_ as a base at 60 °C under aerobic conditions for 24 h.

### 2.2. Scope and Limitations of Substrates

Encouraged by the efficiency of the reaction protocol described above, we investigated the substrate scope. A variety of aromatic terminal alkynes were tested to afford the corresponding aromatic 1,4-disubstituted-1,3-diyne derivatives in good to excellent yields under the optimized conditions. The results are summarized in Table 3. As can be seen, the homocoupling reactions of various aromatic acetylenes with electron-donating groups on aromatic rings such as methyl, *n*-butyl, *tert*-butyl, and methoxy gave almost the same high yields (82%–93%) (Table 3, Entries 2–7). Besides, no significant difference was observed in yield at the same reaction conditions when the effect of different position of the substituent groups on aromatic rings on the homocoupling reaction of aromatic terminal alkynes was studied (Table 3, Entries 2,3,6,7). However, the homocoupling product was obtained in lower yield (56%) when aromatic acetylene with the electron-withdrawing fluoro group on the aromatic ring was homocoupled under optimized conditions (Table 3, Entry 8).

## 3. Experimental Section

### 3.1. Reagents and Machine

The Pd(II) complex catalyst **1** was prepared according to a procedure found in the literature [27]. Aromatic alkyne derivatives were obtained commercially from J&K Chemical Technology (Shanghai, China). All reagents employed in the reaction were analytical grade, and other chemicals were obtained commercially and used without any prior purification. All products were isolated using thin-layer chromatography (Qingdao Haiyang Chemical CO., Ltd, Qingdao, China) with GF254 silica gel using Petroleum ether and ethyl acetate unless otherwise noted. Products described in the literature were characterized using ^1^H-NMR and ^13^C-NMR spectra and compared with previously reported data. ^1^H-NMR and ^13^C-NMR spectra were recorded with a Bruker Avance II 400 spectrometer (Fällanden, Switzerland) using tetramethylsilane as the internal standard and CDCl_3_ as the solvent.

### 3.2. General Experimental Procedure for the Homocoupling Reaction of Various Aromatic Alkynes

All reactions were carried out under aerobic conditions. A mixture of aromatic alkyne (1.0 mmol), AgNO_3_ (0.075 mmol), Cs_2_CO_3_ (1.0 mmol), catalyst compound **1** (0.5 mol %), and *n*-butyl alcohol/H_2_O (in 1:2 proportion, 3 mL) was stirred at 60 °C for 24 h and then extracted three times with ethyl acetate (3 × 15 mL). The combined organic phase was dried with MgSO_4_, filtrated, and then solvent was removed on a rotary evaporator. The product was isolated by thin-layer chromatography. The purified products were identified by ^1^H-NMR and ^13^C-NMR spectroscopy (Figures S1–S10).

### 3.3. Analytical Data of Representative Products

*1,4-Diphenylbuta-1,3-diyne* (Table 3, Entry 1): White solid (m.p. = 86–87 °C, lit [29] 85–86 °C). ^1^H-NMR (400 MHz, CDCl_3_): δ 7.75–7.60 (m, 4H), 7.60–7.40 (m, 6H). ^13^C-NMR (101 MHz, CDCl_3_): δ 138.17, 134.90, 134.13, 127.43, 87.29, 79.68.

*1,4-Di-o-tolybuta-1,3-diyne* (Table 3, Entry 2): White solid (m.p. = 72–74 °C, lit [30] 72–74 °C). ^1^H-NMR (400 MHz, CDCl_3_): δ 8.41 (d, *J* = 7.6 Hz, 2H), 8.19–8.09 (m, 4H), 8.05 (t, *J* = 7.4 Hz, 2H), 3.40 (s, 6H). ^13^C-NMR (101 MHz, CDCl_3_): δ 146.23, 137.53, 134.20, 133.74, 130.29, 126.33, 85.81, 82.22, 25.35.

*1,4-Di-m-tolybuta-1,3-diyne* (Table 3, Entry 3): White solid (m.p. = 69–71 °C, lit [30] 68–70 °C). ^1^H-NMR (400 MHz, CDCl_3_): δ 8.40 (d, *J* = 6.4Hz, 4H), 8.28 (ddd, *J* = 22.5, 9.9, 4.4Hz, 4H), 3.40 (s, 6H). ^13^C-NMR (101 MHz,CDCl_3_): δ144.08, 138.89, 136.05, 135.53, 134.25, 127.54 , 87.56, 79.61, 27.12 .

*1,4-Bis(4-butylphenyl)buta-1,3-diyne* (Table 3, Entry 4): White solid (m.p. = 65–66 °C, lit [31] 67 °C). ^1^H-NMR (400 MHz, CDCl_3_): δ 8.12–7.99 (m, 4H), 7.76 (d, *J* = 7.1 Hz, 4H), 3.22 (t, *J* = 7.6 Hz, 4H), 2.30–2.11 (m, 4H), 1.96 (dd, *J* = 14.0, 7.0 Hz, 4H), 1.55 (t, *J* = 7.3, 1.8 Hz, 6H). ^13^C-NMR (101 MHz, CDCl_3_): δ 148.03, 136.00, 132.16, 122.59, 85.20, 77.18, 39.30, 36.92, 25.94, 17.54.

*1,4-Bis(4-tert-butylphenyl)buta-1,3-diyne* (Table 3, Entry 5)*:* White solid (m.p. = 202–204 °C, lit [32] 203–204 °C). ^1^H-NMR (400 MHz, CDCl_3_): δ 7.88 (d, *J* = 7.4 Hz, 4H), 7.77 (d, *J* = 7.5 Hz, 4H), 1.73 (s, 18H). ^13^C-NMR (101 MHz, CDCl_3_): δ 156.51, 136.24, 129.45, 122.80, 85.49, 77.50, 38.86, 35.08.

*1,4-Bis(4-methoxyphenyl)buta-1,3-diyne* (Table 3, Entry 6): White solid (m.p. = 39–141 °C, lit [29] 138–139 °C). ^1^H-NMR (400 MHz, CDCl_3_): δ 8.17 (d, *J* = 8.6 Hz, 4H), 7.56 (d, *J* = 8.6 Hz, 4H), 4.51 (s, 6H). ^13^C-NMR (101 MHz, CDCl_3_): δ 164.19, 137.99, 136.34, 133.18, 118.09, 117.85, 85.51, 85.21, 76.92, 59.27.

*1,4-Bis(2-methoxyphenyl)buta-1,3-diyne* (Table 3, Entry 7): White solid(m.p. = 72–74 °C, lit [13] 72–74 °C). ^1^H-NMR (400 MHz, CDCl_3_): δ 7.73 (dd, *J* = 7.6, 1.3 Hz, 2H), 7.62–7.52 (m, 2H), 7.15 (dd, *J* = 16.6, 8.1 Hz, 4H), 4.14 (s, 6H). ^13^C-NMR: δ 163.25, 136.28, 132.53, 122.43, 113.11, 112.60, 80.63, 79.91, 57.71.

*1,4-Bis(4-fluorophenyl)buta-1,3-diyne* (Table 3, Entry 8): White solid (m.p. = 190–192 °C, lit [29] 192–193 °C). ^1^H-NMR (400 MHz, CDCl_3_): δ 8.09 (dd, *J* = 8.6, 5.4 Hz, 4H), 7.62 (t, *J* = 8.6 Hz, 4H). ^13^C-NMR (101 MHz, CDCl_3_): δ 137.11, 118.60, 118.38, 83.00, 76.09.

*1,4-Bis(3-chlorophenyl)buta-1,3-diyne* (Table 3, Entry 9): White solid (m.p. = 73–74 °C, lit [33] 73 °C). ^1^H-NMR (400 MHz, CDCl_3_): δ 7.86 (s, 2H), 7.74 (dd, *J* = 19.5, 8.0 Hz, 4H), 7.63 (dd, *J* = 10.7, 5.0 Hz, 2H). ^13^C-NMR (101 MHz, CDCl_3_): δ 137.61, 135.52, 133.92, 132.98, 126.53, 83.82, 77.96.

*1,4-Bis(2-bromophenyl)buta-1,3-diyne* (Table 3, Entry 10)*:* White solid (m.p. = 180–182 °C, lit [34] 182 °C). ^1^H-NMR (400 MHz, CDCl_3_): δ 7.68–7.55 (m, 4H), 7.38–7.20 (m, 4H). ^13^C-NMR (101 MHz, CDCl_3_): δ 138.78, 136.84, 134.65, 133.15, 132.01, 131.37, 131.33, 131.28, 130.44, 128.28, 85.32, 82.09.

## 4. Conclusions

In summary, we have developed a new and efficient Pd(II)/AgNO_3_ catalytic system for the homocoupling of various terminal alkynes. It is noteworthy that our protocol employs a relatively low-palladium catalyst loading in aqueous media under aerobic conditions to obtain the coupled products in good to excellent yields. Currently, further efforts to study the mechanism and apply the new approach in other transformations are under way in our laboratory.

## Figures and Tables

**Table 1:** Effects of solvent and base on the homocoupling of phenylacetylene ^a^.

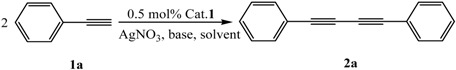

Entry	Ase	Solvent	Yield ^c^ (%)
1 ^b^	NaOH	THF/H_2_O	10
2	NaOH	THF/H_2_O	51
3	KOH	THF/H_2_O	45
4	Na_2_CO_3_	THF/H_2_O	67
5	K_2_CO_3_	THF/H_2_O	57
6	NaHCO_3_	THF/H_2_O	63
7	NaH_2_PO_4_	THF/H_2_O	71
8	KHCO_3_	THF/H_2_O	64
9	KH_2_PO_4_	THF/H_2_O	70
10	K_3_PO_4_	THF/H_2_O	70
11	Cs_2_CO_3_	THF/H_2_O	85
12	NaF	THF/H_2_O	47
13	CH3COONa	THF/H_2_O	53
14	NEt_3_	THF/H_2_O	63
15	Pyridine	THF/H_2_O	54
16	Cs_2_CO_3_	DMSO/H_2_O	47
17	Cs_2_CO_3_	*N*,*N*-Dimethylacetylamide/H_2_O	39
18	Cs_2_CO_3_	PEG400/H_2_O	59
19	Cs_2_CO_3_	Acetone/H_2_O	88
20	Cs_2_CO_3_	1,4-Dioxane/H_2_O	79
21	Cs_2_CO_3_	Ethanol/H_2_O	54
22	Cs_2_CO_3_	*N*-Butyl alcohol/H_2_O	93
23	Cs_2_CO_3_	Methanol/H_2_O	45

^a^ The reaction was performed with phenylacetylene (1 mmol), Pd(II) complex catalyst **1** (0.5 mol %), AgNO_3_ (0.05 mmol), and base (1 mmol) in solvent/H_2_O (2.5 mL, *v*/*v* = 4:1) at 60 °C under aerobic conditions for 24 h. ^b^ In the absence of AgNO_3_. ^c^ Isolated yield.

**Table 2:** Effects of other reaction conditions on the homocoupling of phenylacetylene ^a^.

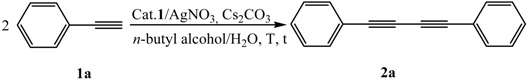

Entry	*n*-Butyl alcohol/H_2_O (*v*/*v*)	Catalyst (mol %)	AgNO_3_ (mmol)	Time (h)	Temperature (°C)	Yield ^b^ (%)
1	3:0	0.5	0.05	24	60	50
2	2:0.5	0.5	0.05	24	60	89
3	2:1	0.5	0.05	24	60	89
4	1.5:1.5	0.5	0.05	24	60	93
5	1:2	0.5	0.05	24	60	97
6	0:3	0.5	0.05	24	60	48
7	1:2	0	0.05	24	60	trace
8	1:2	0.25	0.05	24	60	65
9	1:2	1	0.05	24	60	96
10	1:2	1.5	0.05	24	60	90
11	1:2	0.5	0	24	60	trace
12	1:2	0.5	0.01	24	60	35
13	1:2	0.5	0.025	24	60	62
14	1:2	0.5	0.075	24	60	99
15	1:2	0.5	0.1	24	60	86
16	1:2	0.5	0.075	4	60	13
17	1:2	0.5	0.075	12	60	72
18	1:2	0.5	0.075	21	60	76
19	1:2	0.5	0.075	30	60	86
20	1:2	0.5	0.075	24	40	56
21	1:2	0.5	0.075	24	80	92

^a^ The reaction was performed with phenylacetylene (1 mmol) and Cs_2_CO_3_ (1 mmol) under aerobic conditions; ^b^ Isolated yield.

**Table 3:** Pd(II)/AgNO_3_-catalyzed the homocoupling reactions of aromatic terminal alkynes ^a^.

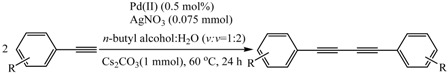

Entry	Alkyne	Product	Yield ^b^ (%)
1	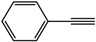	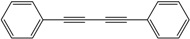	99
2	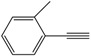	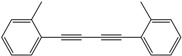	86
3	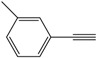	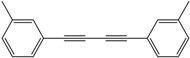	84
4	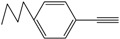	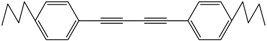	88
5	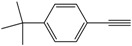	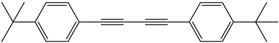	82
6	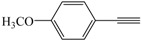	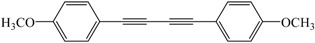	83
7	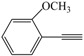	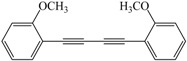	93
8	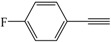	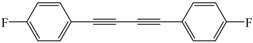	56
9	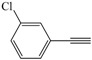	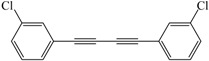	78
10	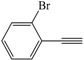	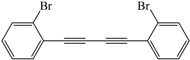	82

^a^ Carried out with aromatic terminal alkyne (1 mmol), Pd(II) complex catalyst **1** (0.5 mol %), AgNO_3_ (0.075 mmol), and Cs_2_CO_3_ (1 mmol) in *n*-butyl alcohol/H_2_O (in 1:2 proportion, 3 mL) at 60 °C under aerobic conditions for 24 h; ^b^ Isolated yield.

## Supplementary Materials

Supplementary materials can be accessed at http://www.mdpi.com/1420-3049/21/5/606/s1.
