# Supplementary material for: The Homocoupling Reaction of Aromatic Terminal Alkynes by a Highly Active Palladium(II)/AgNO3 Cocatalyst in Aqueous Media Under Aerobic Conditions
Source: Molecules. 2016 May 10;21(5):606. doi: 10.3390/molecules21050606 (PMC6274053; doi:10.3390/molecules21050606)
Supplement: Supplementary file 1 [file molecules-21-00606-s001.pdf]

Supplementary Materials: Homocoupling Reaction of Aromatic Terminal Alkynes by a Highly Active Palladium(II)/AgNO3 Cocatalyst in Aqueous Media Under Air

Mengping Guo, Bo Chen, Meiyun Lv, Xiuling Zhou, Yongju Wen and Xiuli Shen

Characterization

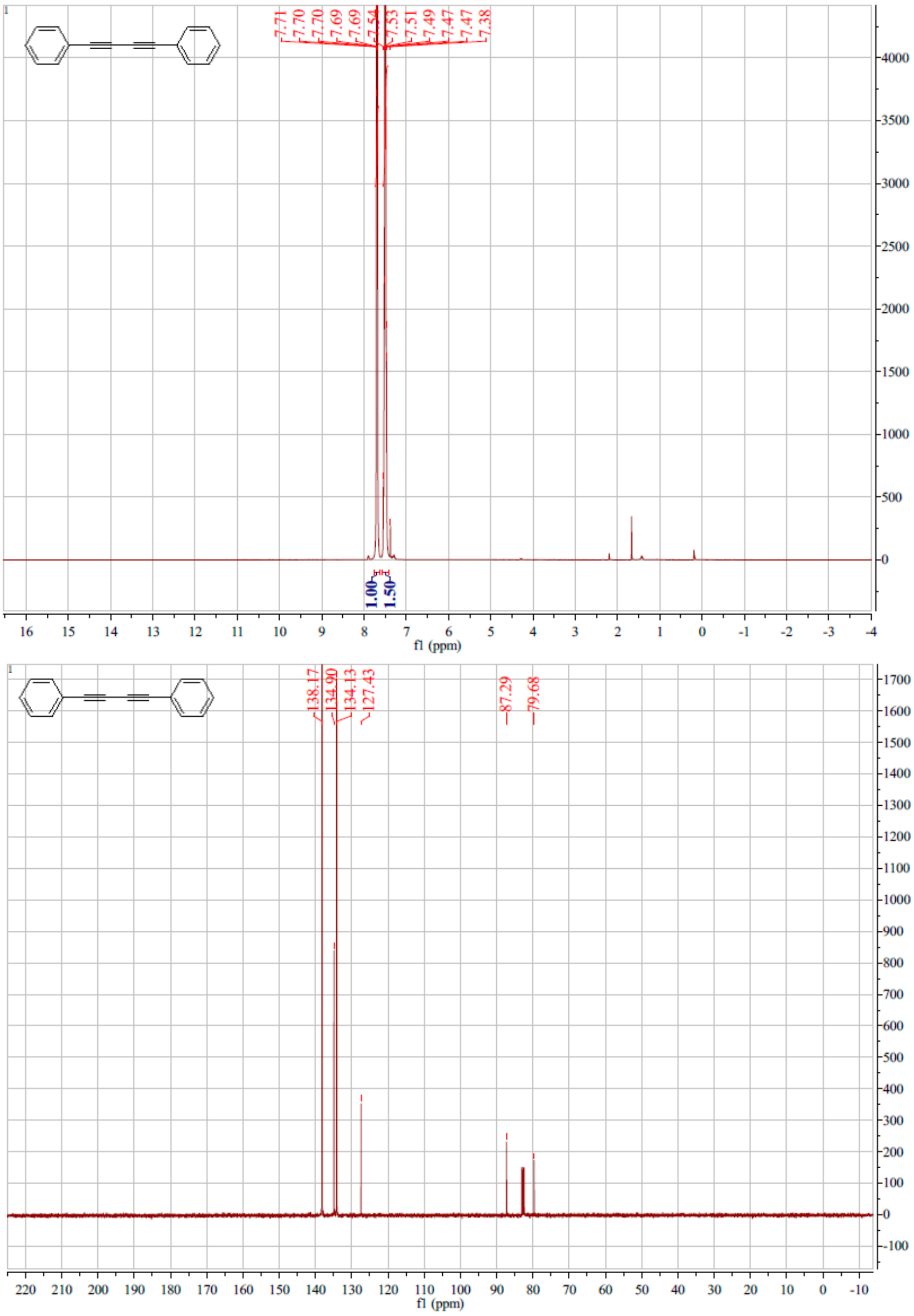

Figure S1. ^{1}H-NMR and ^{13}C-NMR of 1,4-diphenylbuta-1,3-diyne.

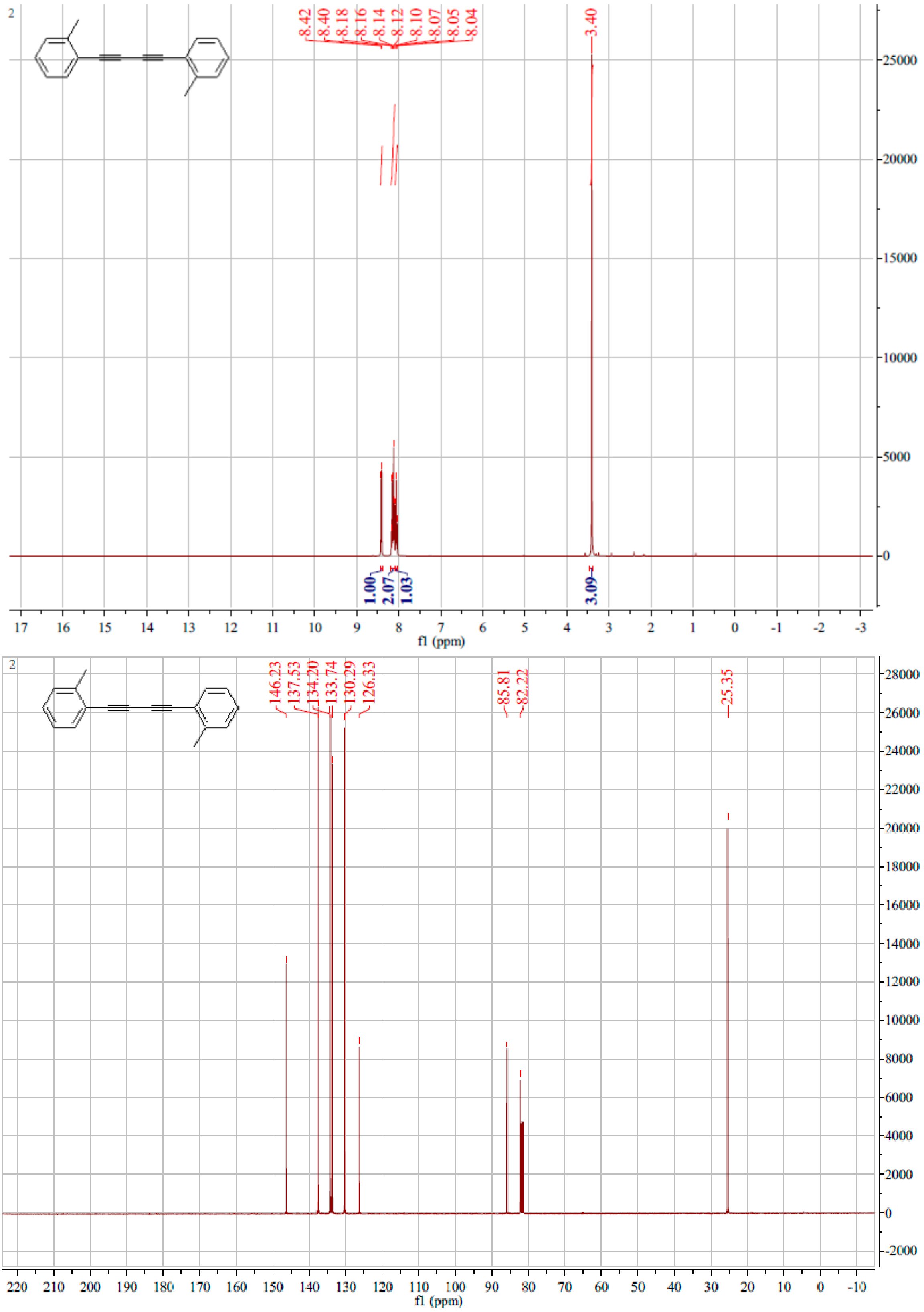

Figure S2. ^{1}H-NMR and ^{13}C-NMR of 1,4-di-*o*-tolybuta-1,3-diyne.

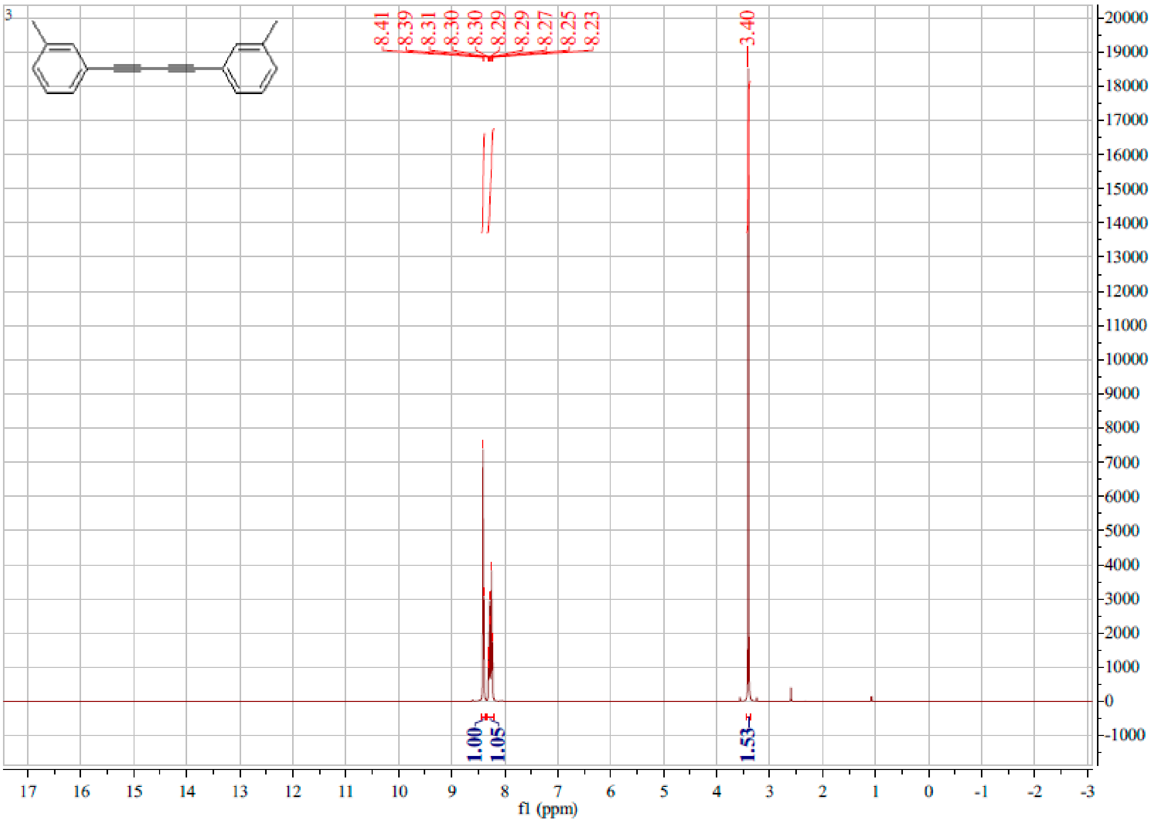

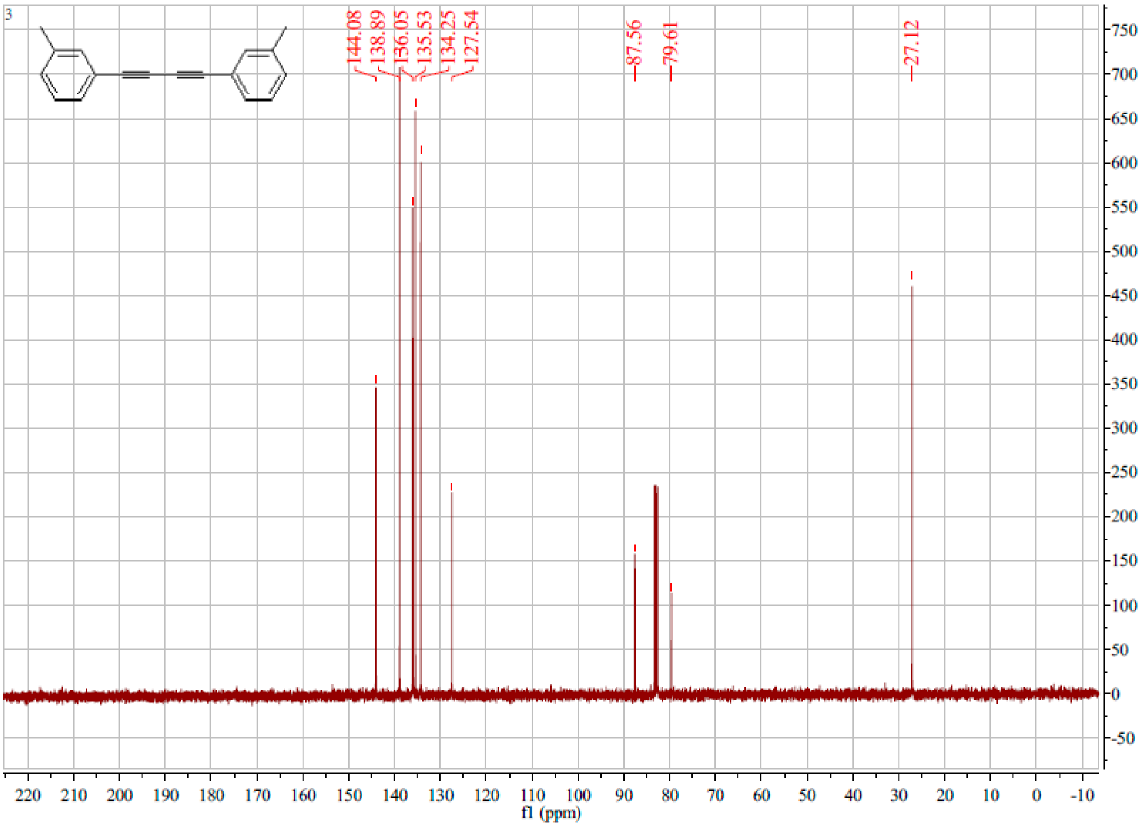

Figure S3. ^{1}H-NMR and ^{13}C-NMR of 1,4-di-*m*-tolybuta-1,3-diyne.

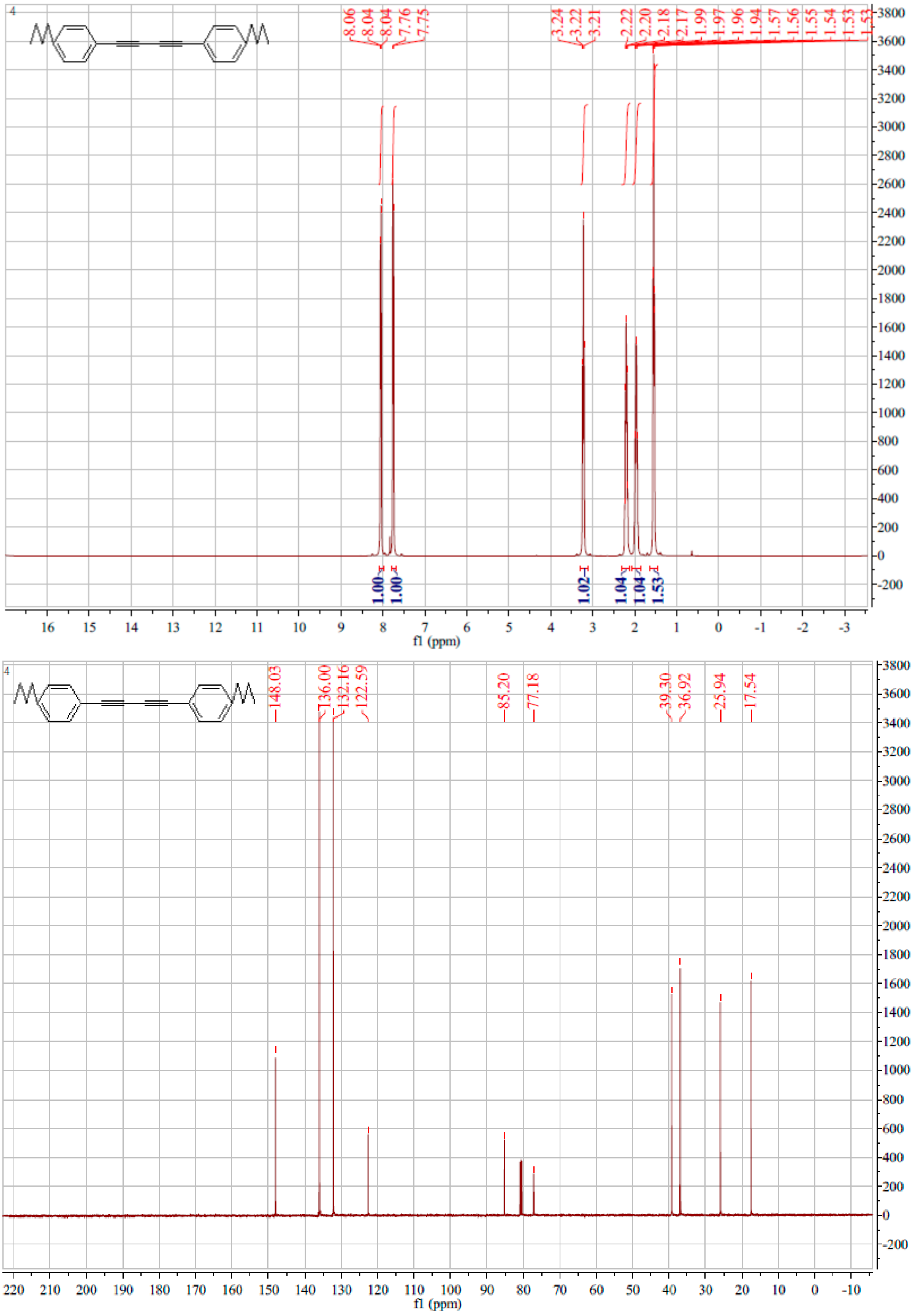

Figure S4. ^{1}H-NMR and ^{13}C-NMR of 1,4-*bis*(4-butylphenyl)buta-1,3-diyne.

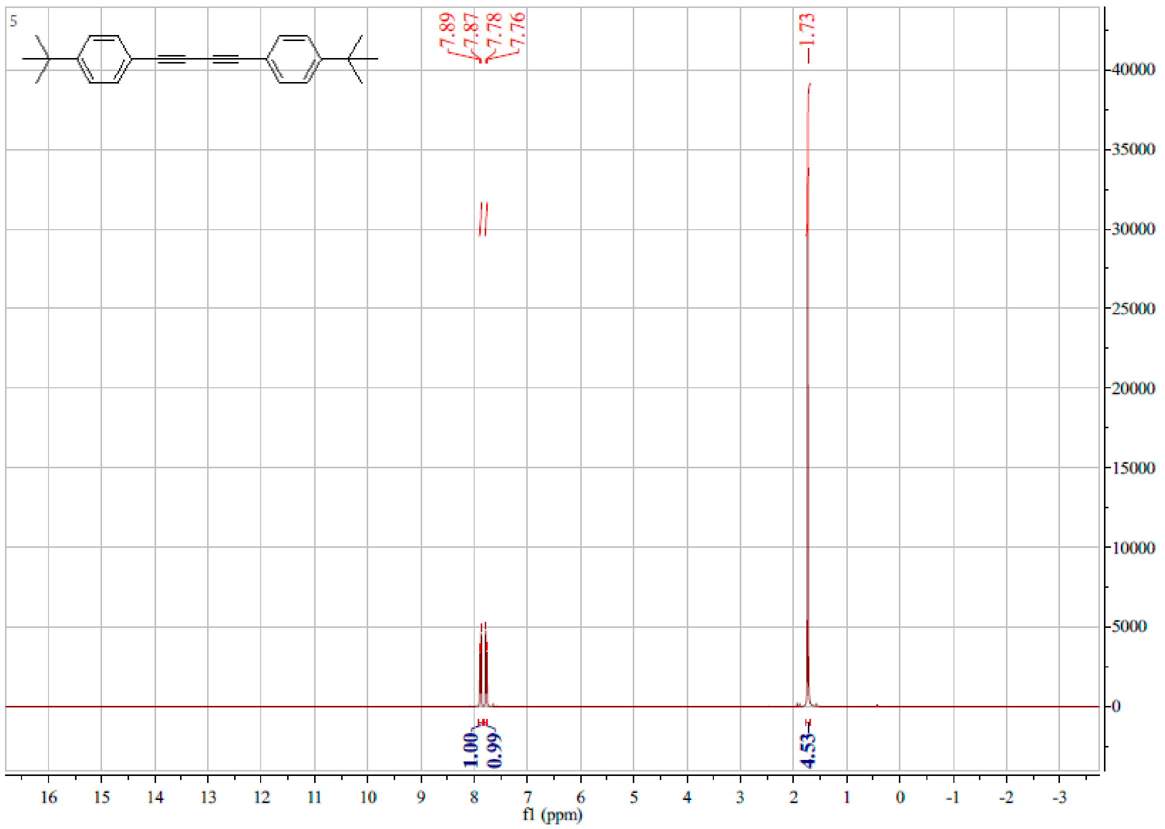

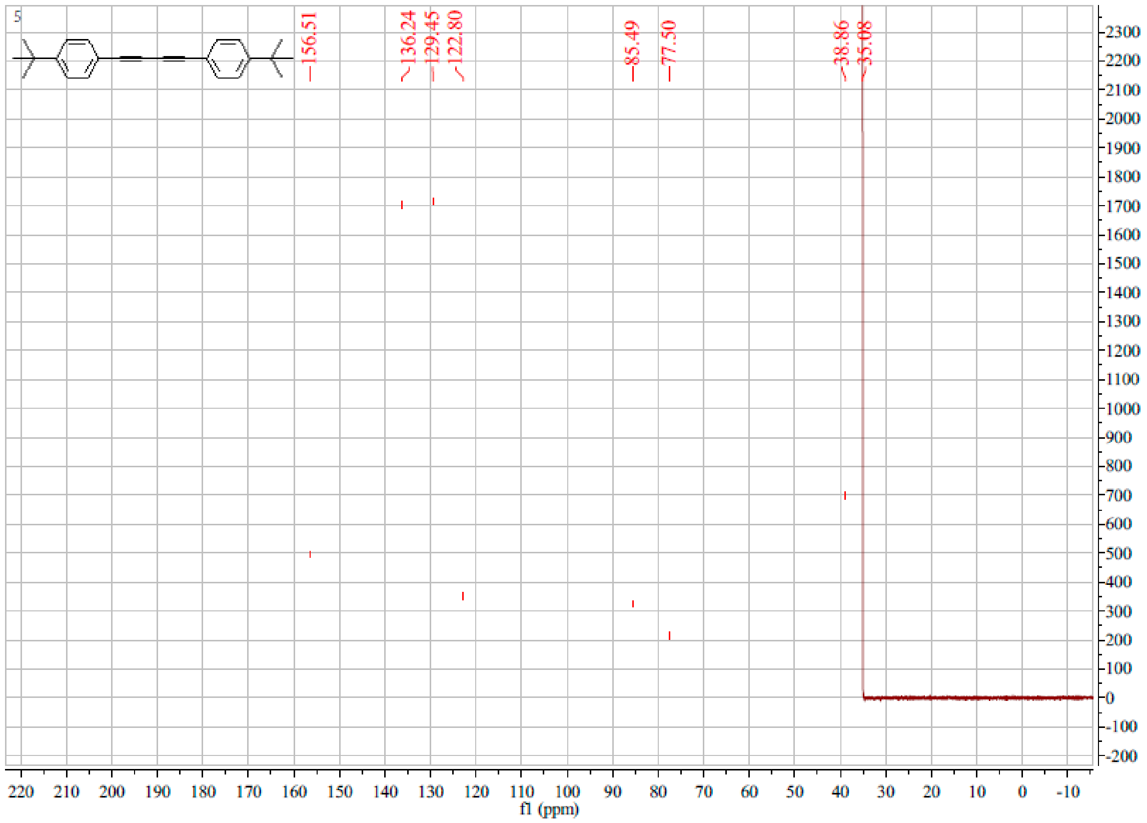

Figure S5. ^{1}H-NMR and ^{13}C-NMR of 1,4-*bis*(4-(tert-butyl)phenyl)buta-1,3-diyne.

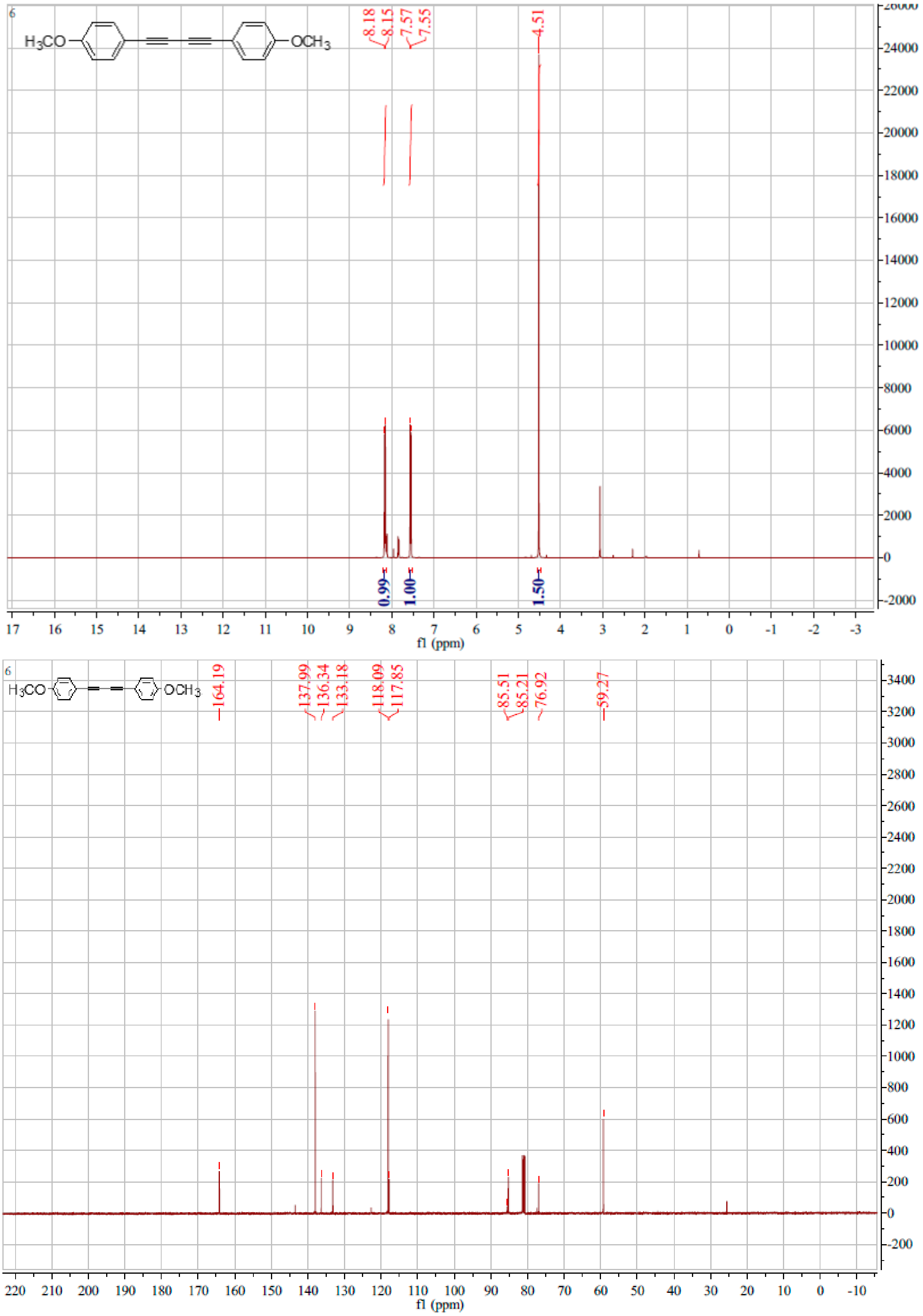

Figure S6. ^{1}H-NMR and ^{13}C-NMR of 1,4-*bis*(4-methoxyphenyl)buta-1,3-diyne.

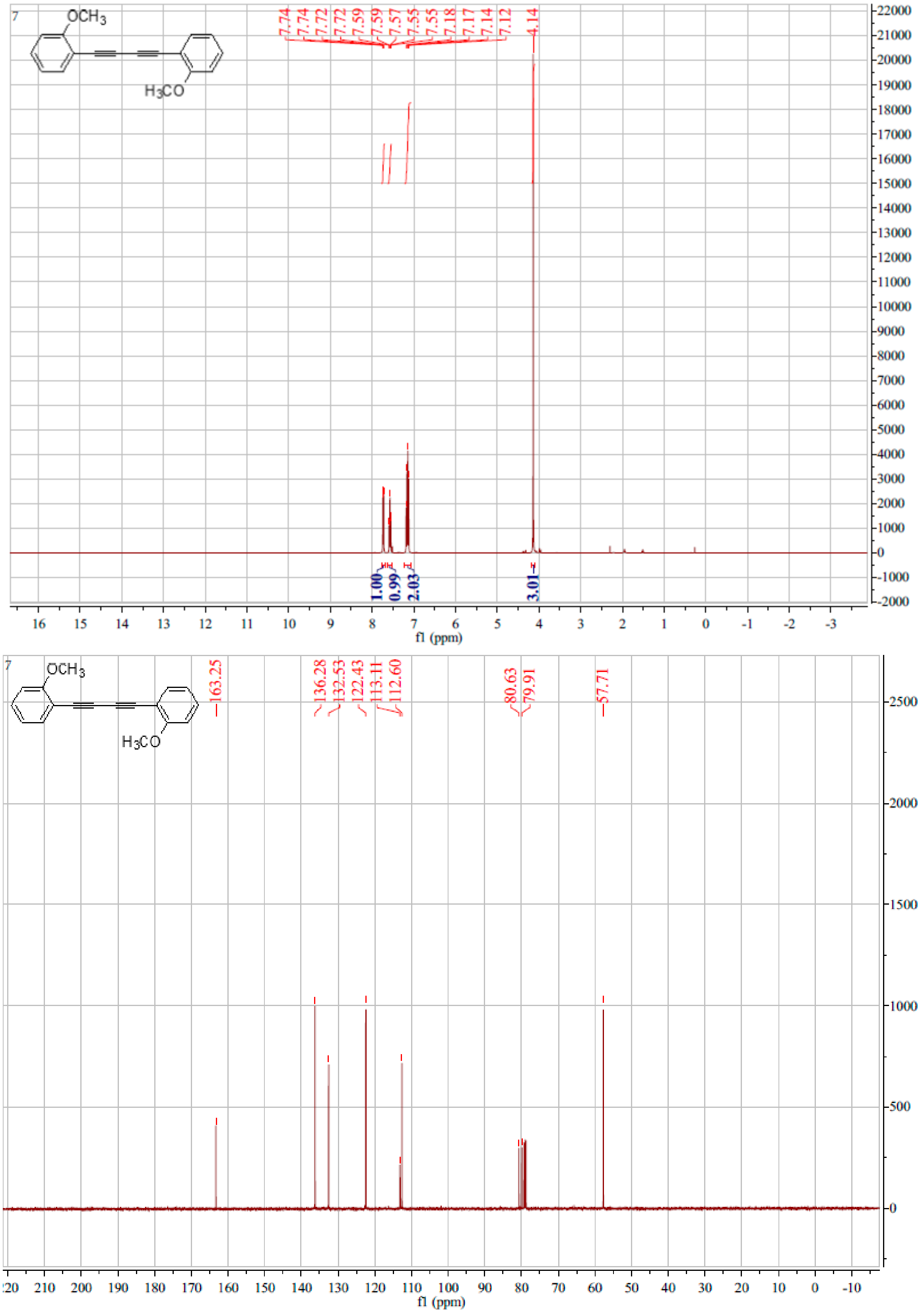

Figure S7. ¹H-NMR and ¹³C-NMR of 1,4-*bis*(3-methoxyphenyl)buta-1,3-diyne

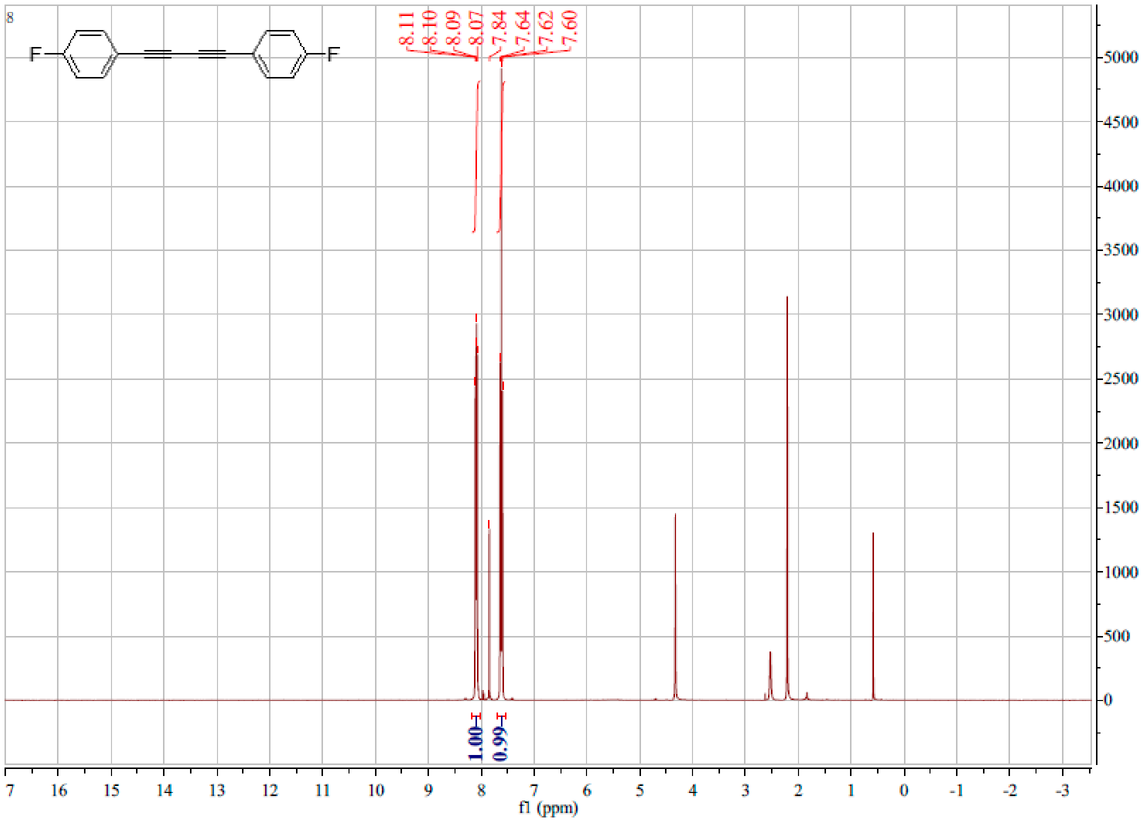

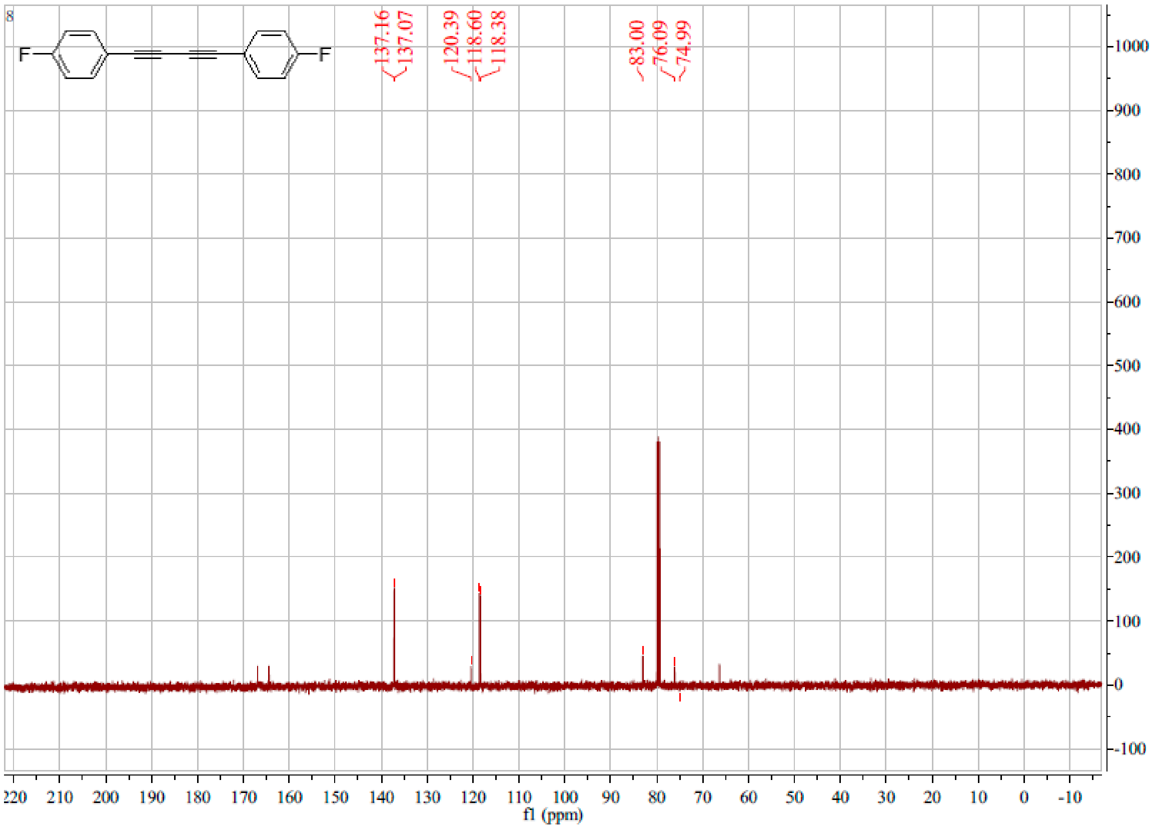

Figure S8. ^{1}H-NMR and ^{13}C-NMR of 1,4-*bis*(4-fluorophenyl)buta-1,3-diyne.

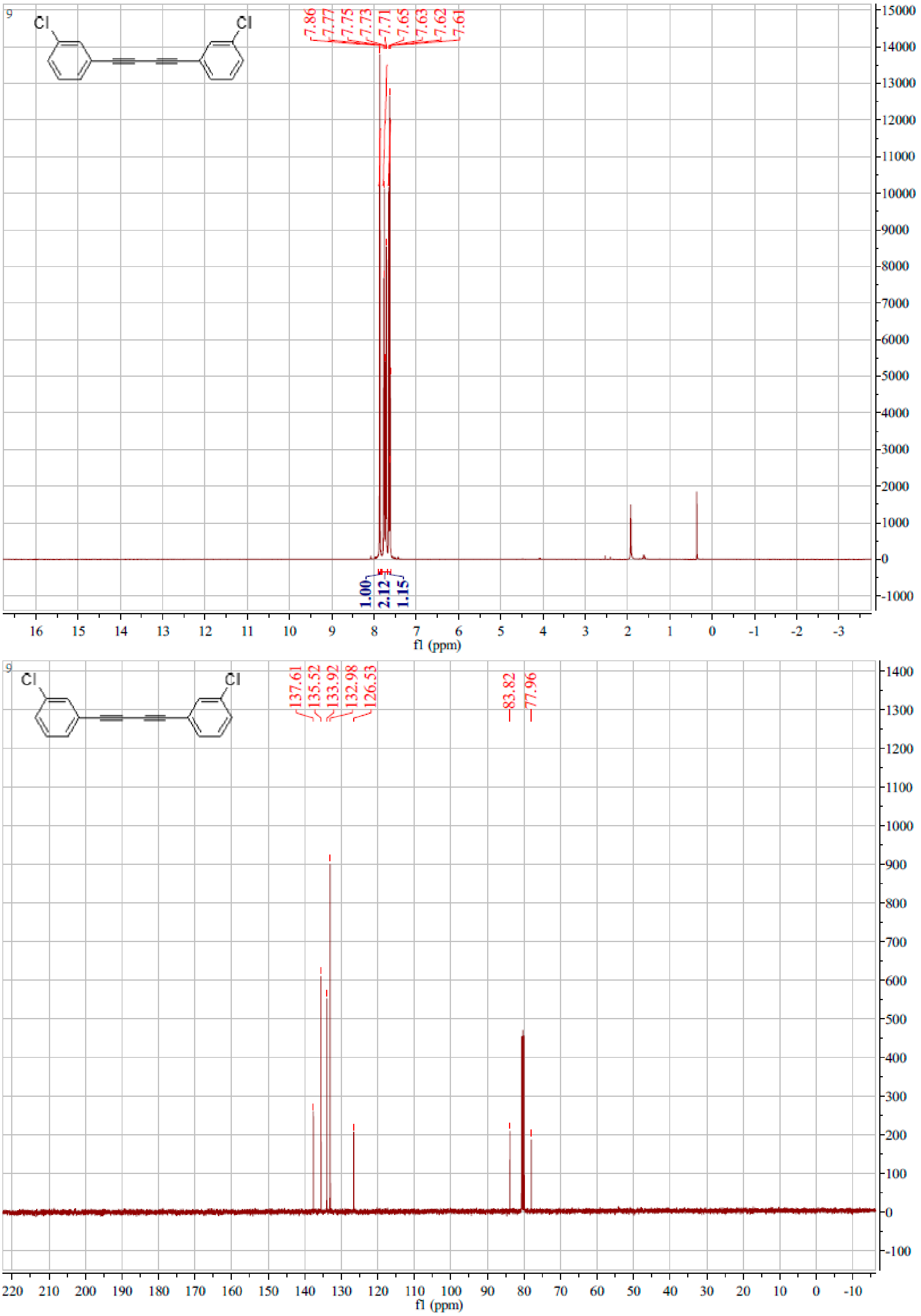

Figure S9. ^{1}H-NMR and ^{13}C-NMR of 1,4-*bis*(3-chlorophenyl)buta-1,3-diyne.

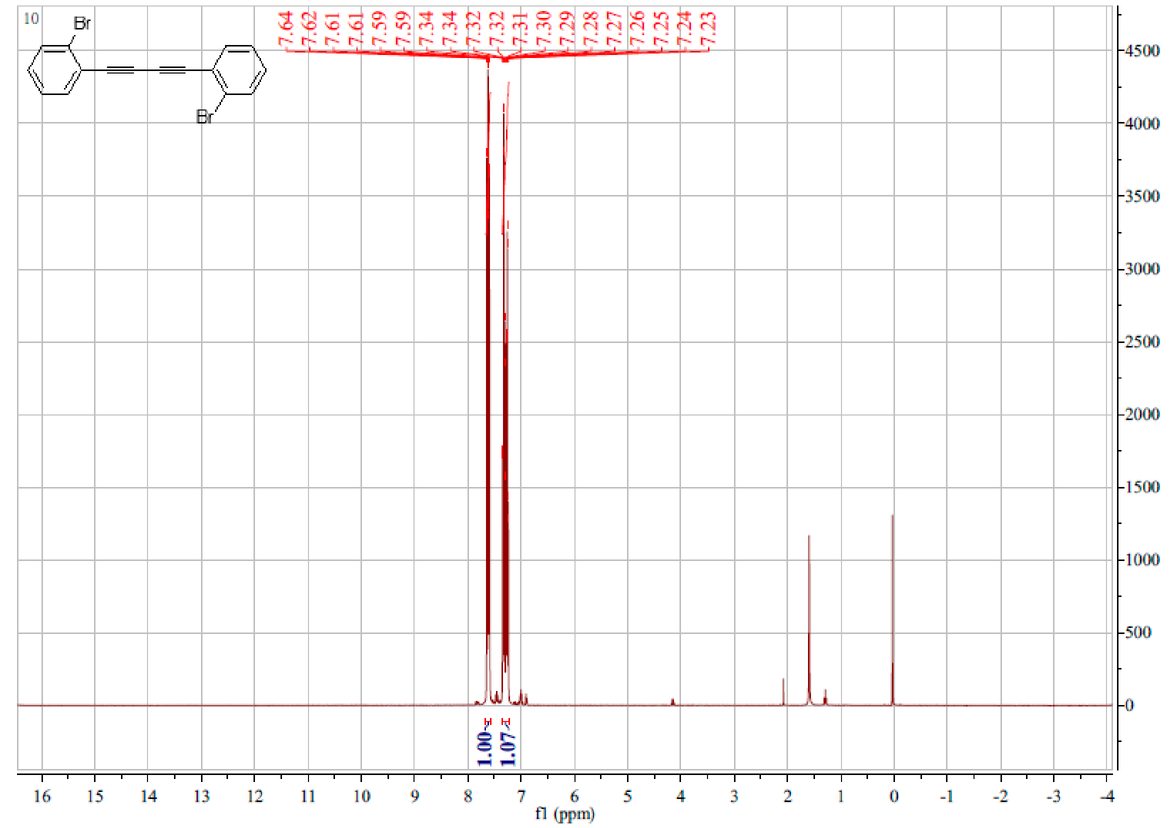

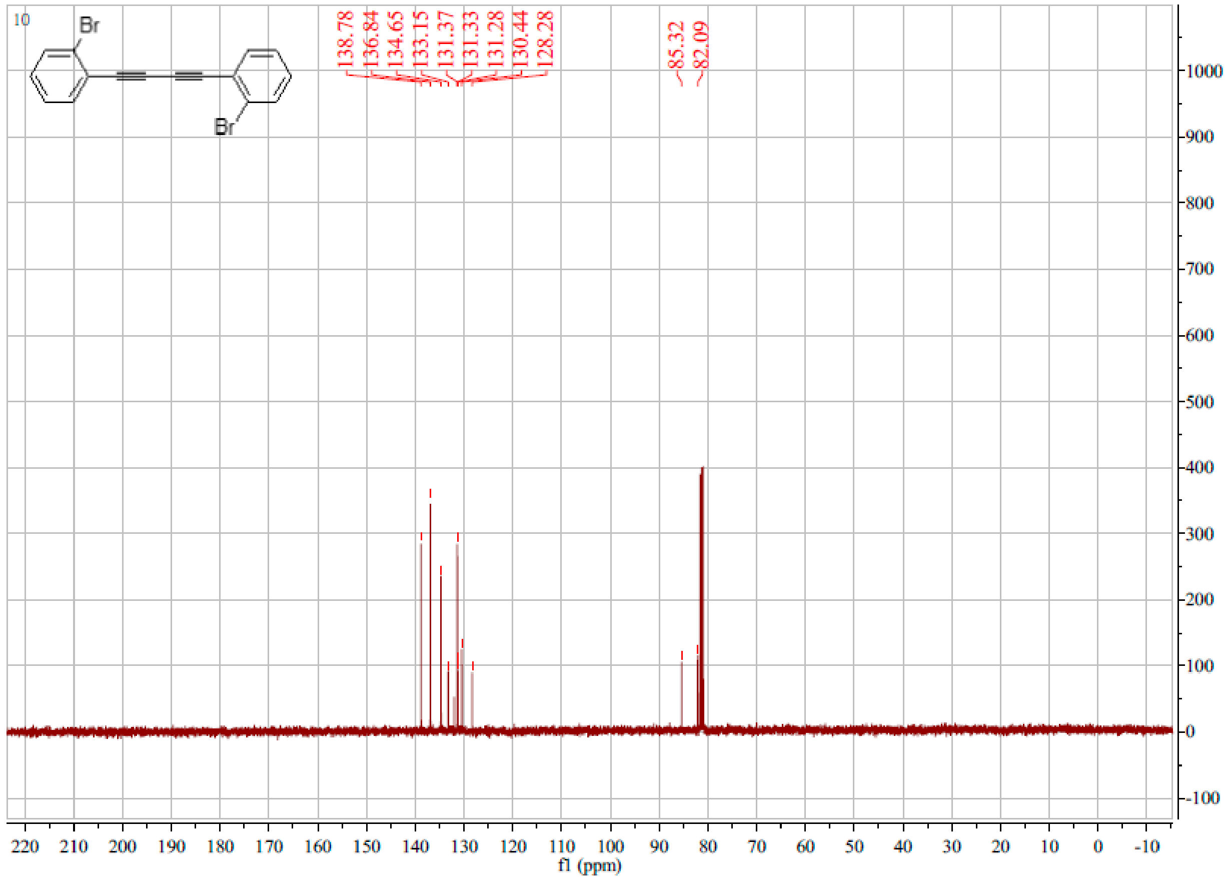

Figure S10. ^{1}H-NMR and ^{13}C-NMR of 1,4-*bis*(3-chlorophenyl)buta-1,3-diyne.
